# Thermionic Emission of Atomic Layer Deposited MoO_3_/Si UV Photodetectors

**DOI:** 10.3390/ma16072766

**Published:** 2023-03-30

**Authors:** Mohamed A. Basyooni, A. E. H. Gaballah, Mohammed Tihtih, Issam Derkaoui, Shrouk E. Zaki, Yasin Ramazan Eker, Şule Ateş

**Affiliations:** 1 Department of Nanotechnology and Advanced Materials, Graduate School of Applied and Natural Science, Selçuk University, Konya 42030, Turkey; 2 Science and Technology Research and Application Center (BITAM), Necmettin Erbakan University, Konya 42090, Turkey; 3 Solar and Space Research Department, National Research Institute of Astronomy and Geophysics (NRIAG), Cairo 11421, Egypt; 4 Photometry and Radiometry Division, National Institute of Standards (NIS), Tersa St, Al-Haram, Giza 12211, Egypt; 5 Institute of Ceramics and Polymer Engineering, University of Miskolc, H-3515 Miskolc, Hungary; 6 Laboratory of Solid-State Physics, Faculty of Sciences Dhar el Mahraz, University Sidi Mohammed Ben Abdellah, P.O. Box 1796, Atlas Fez 30000, Morocco; 7 Theoretical Physics Department, National Research Center, Dokki, Cairo 12622, Egypt; 8 Department of Metallurgy and Material Engineering, Faculty of Engineering and Architecture, Necmettin Erbakan University, Konya 42060, Turkey; 9 Department of Physics, Faculty of Science, Selçuk University, Konya 42075, Turkey

**Keywords:** MoO_3_, electric and optoelectronics, ultrathin films, thermionic emission, UV illuminations

## Abstract

Ultrathin MoO_3_ semiconductor nanostructures have garnered significant interest as a promising nanomaterial for transparent nano- and optoelectronics, owing to their exceptional reactivity. Due to the shortage of knowledge about the electronic and optoelectronic properties of MoO_3_/*n*-Si via an ALD system of few nanometers, we utilized the preparation of an ultrathin MoO_3_ film at temperatures of 100, 150, 200, and 250 °C. The effect of the depositing temperatures on using bis(tbutylimido)bis(dimethylamino)molybdenum (VI) as a molybdenum source for highly stable UV photodetectors were reported. The ON–OFF and the photodetector dynamic behaviors of these samples under different applied voltages of 0, 0.5, 1, 2, 3, 4, and 5 V were collected. This study shows that the ultrasmooth and homogenous films of less than a 0.30 nm roughness deposited at 200 °C were used efficiently for high-performance UV photodetector behaviors with a high sheet carrier concentration of 7.6 × 10^10^ cm^−2^ and external quantum efficiency of 1.72 × 10^11^. The electronic parameters were analyzed based on thermionic emission theory, where Cheung and Nord’s methods were utilized to determine the photodetector electronic parameters, such as the ideality factor (*n*), barrier height (Φ_0_), and series resistance (R_s_). The *n*-factor values were higher in the low voltage region of the I–V diagram, potentially due to series resistance causing a voltage drop across the interfacial thin film and charge accumulation at the interface states between the MoO_3_ and Si surfaces.

## 1. Introduction

Ultrathin films are thin films with thicknesses of a few nanometers, making them ultra-compact and highly reactive [1,2,3,4]. These films possess exceptional properties, including a high surface area, electrical conductivity, and optical transparency, which make them ideal for applications in electronics, ferromagnetism, catalytic, optics, and energy storage [1,3,5,6,7]. Molybdenum trioxide (MoO_3_) is a metal oxide with several interesting properties, making it a popular material for the fabrication of thin films and ultrathin films. MoO_3_ is an interesting material to study due to its distinctive electrical and optical characteristics [8,9]. As an *n*-type semiconductor, it has high electron mobility, making it practical for use in electronic devices such as energy storage devices, transistors, solar cells, and supercapacitors [5,10,11]. Additionally, MoO_3_ also serves as a good gas sensor [12] and can detect various gases, including oxygen and nitrogen oxides. The combination of optical transparency, high conductivity, wide bandgap, strong electro-optical properties, and environmental stability make α-MoO_3_ an attractive material for use in optoelectronic devices. For example, α-MoO_3_ is transparent in the visible light range, which makes it a suitable material for optoelectronic applications such as transparent displays, touch screens, and solar cells. The high electrical conductivity and wide bandgap of α-MoO_3_ make it a useful material in electronic devices such as lithium-ion batteries, electrocatalysts, a hole-transporting layer, gate dielectrics, and light-emitting diodes [13]. The environmental stability of α-MoO_3_ makes it a useful material in optoelectronic and gas sensor devices that are expected to have a long operational lifetime [14].

The atomic scale of MoO_3_ thin film is prepared with many methods such as atomic layer etching and ALD [15]. However, for Optoelectronic-devices-based silicon substrates, ALD is more interesting [15]. ALD is a method that is well-suited for the growth of ultrathin film models. This method utilizes self-limiting interactions between gaseous precursors and surfaces to achieve precise control over the thickness and composition of thin films. Additionally, ALD can also provide control over the texture and topography of the films in some cases. Mo(CO)_6_ and oxygen (O_2_) plasma have been used to deposit MoO_3_ ultrathin film on sapphire for MoS_2_ optimization [16,17,18]. Additionally, researchers have explored the synthesis of 2D MoS_2_ and WS_2_ from transition metal oxides (TMOs) using different metal–organic precursors. The 2D MoS_2_ is created by sulfurizing MoO_3_ film deposited using bis(tert-butylimido)bis(dimethylamino) molybdenum ((tBuN)_2_(NMe_2_)_2_Mo) and O_3_ through thermal ALD at a temperature of 300 °C, or using the same precursor and remote O_2_ plasma through plasma-enhanced ALD at 150 °C. By optimizing the ALD process parameters such as the growth temperature, precursor exposure time, and surface preparation, a uniform and controllable 2D MoS_2_ film can be produced [19]. MoO_x_ thin film was also produced by ALD via depositing of a(NtBu)_2_(NMe_2_)_2_Mo precursor with O_2_ plasma at a temperature of 50° C. The films were found to be sub-stoichiometric [20].

A semiconductor photodetector is a device that utilizes a semiconductor material as an active layer for absorbing light and converting it into an electrical signal. Depending on the properties of the semiconductor material, photodetectors can be designed to operate within specific spectral ranges. The visible-to-infrared (IR) spectral range is dominated by Si-based technology, which is widely used in commercial applications. However, the detection of the ultraviolet (UV) range has also been made possible by implementing filters in Si-based photodetectors [21]. Mendoza and colleagues created the first photodetector on a large scale using MoO_3_ as the active material. The MoO_3_ layer was synthesized through van der Waals epitaxy on muscovite mica and had a thickness ranging from 1.4 to 6 nm. After being created, the layer was transferred to a SiO_2_ substrate. The field-effect device produced a responsivity of 30 mA/W when tested at a wavelength of 405 nm, but exhibited a relatively slow response time of around 20 s [22]. In another study, Li and their team demonstrated a UV photodetector made from centimeter-scale MoO_3_ single crystals grown through physical vapor deposition (PVD). The photodetector showed a high responsivity of 65.6 A/W at 405 nm after undergoing a 2-h vacuum annealing process. As a result of annealing, the mobility of the photogenerated carriers was enhanced, leading to a faster response time of 2 s [23]. Arash et al. developed high-performance ultraviolet photodetectors by employing non-stoichiometric MoO_3-x_ layers. The presence of oxygen vacancies created partially reduced the Mo^5+^ states, which acted as electron traps, facilitating the separation of charge carriers. The photodetector demonstrated a high responsivity of 54.4 A/W at 365 nm and exhibited a fast response time of 200 microseconds [24]. In another study, Zheng et al. created a flexible ultraviolet photodetector on single crystalline MoO_3_ nanosheets grown through physical vapor deposition (PVD). The flexible photodetector maintained its performance despite its flexibility, exhibiting a maximum responsivity of 524 mA/W and an on/off ratio of over two orders under ultraviolet illumination at 380 nm. However, the slower response time of 0.94 s was attributed to the presence of interface states between the MoO_3_ and the flexible polyethylene terephthalate (PET) substrate [25]. In a unique research project, Hu and their colleagues studied the anisotropic photo response of few-nanometer-thick MoO_3_ single crystals that were grown through a vapor–solid process. They found that the calculated responsivity was 67.9 and 6.1 A/W along the b and c axes, respectively. The researchers attributed the anisotropic photo response to differences in the effective mass and carrier mobility along the two crystal axes [26]. Furthermore, MoO_3_ is still interesting for visible optoelectronics. A photodetector based on a single MoO_3_ nanobelt with a wide visible spectrum response was created by Wei Chen et al. The pristine MoO_3_ nanobelt had low conductivity and no photoresponse for nearly all visible lights. Under the illumination of 680 nm light, the photodetector showed a high responsivity of 56 A/W and external quantum efficiency of 102% [27]. However, the fabrication of MoO_3_ by ALD for optoelectronics is still lacking. Recently, Basyooni et al. prepared an ultrathin MoO_3_ using ALD for UV photonic applications. The results showed that the film thickness increased with an increasing deposition temperature. According to the authors, the films that were grown at a temperature of 150 °C showed the greatest suitability for use in UV optoelectronic applications, as they exhibited high stability even when subjected to low applied bias voltages [28].

In this study, due to the shortage of knowledge about the electronic and optoelectronic properties of MoO_3_/*n*-type Si via an ALD system with a thickness of a few nanometers, we utilized the preparation of a few-nanometers-scale MoO_3_ thin film on an *n*-Si substrate at temperatures of 100, 150, 200, and 250 °C. The effect of the depositing temperatures using a bis(t-butylimido)bis(dimethylamino)molybdenum (VI) as a molybdenum source for highly stable UV photodetectors was reported. The electrical parameters of the photodetector in the dark and under UV illuminations in the study were analyzed based on the thermionic emission theory. Namely, two methods, Cheung and Nord’s, were employed to calculate the photodetector’s electronic parameters, including the ideality factor (*n*), barrier height (Φ_0_), and series resistance (R_s_). The ON–OFF and the photodetector dynamic behaviors of these samples under different applied voltages of 0, 0.5, 1, 2, 3, 4, and 5 V were collected. The photodetector stability with time at different applied voltages of 0, 0.5, 1, 2, 3, 4, and 5 V for both the dark and UV illumination conditions were tested for each sample. In addition, the performances of these samples were tested as a UV photodetector while calculating the responsivity, EQE, detectivity, photocurrent gain, and response/recovery time of these samples.

## 2. Materials and Methods

### 2.1. Preparing Ultrathin MoO_3_ Film

The preparation of a MoO_3_ thin film using ALD (AT410 ALD system—ANRIC Technologies, Billerica, MA, USA) was described previously [28]. Briefly, the samples were deposited with Ozone source (ATO_3_ ozone system—ANRIC Technology, Toronto, ON, Canada) as an oxidizing source and bis(t-butylimido)bis(dimethylamino)molybdenum (VI) as the molybdenum (Strem Chemicals, Newburyport, MA, USA) source [29,30,31]. It possesses favorable properties for ALD such as stability in high heat, sample vapor pressure, and reactiveness during typical ALD processing temperatures. We kept the number of pulses constant at 100 pulses and changed the deposition temperature of the substrate. We used Silicon as a substrate in this study. We performed a deposition at 100, 150, 200, and 250 °C, and these samples are denoted as S1, S2, S3, and S4. After that, we made a post-annealing process at 600 °C for 15 min to make the films more crystalline and utilized the α-MoO_3_ as reported before [15,32]. Finally, a silver past was used as conductive electrodes connected to the probe station.

### 2.2. Characterization Methods

The film deposition thickness was optimized and verified using a Filmetrics F20 Thin Film Analyzer. The roughness values were calculated using the XEI 4.3.4 2016 data processing and analysis software, after atomic force microscopy (AFM) was conducted in contact mode over a scanning area of 5 × 5 µm at a tip scan speed of 1 Hz, using a Park XE7 system. The ZEISS GeminiSEM 500 field emission scanning electron microscope (FESEM) was used to observe the surface morphology, while an Oxford Ultime Extreme ZEISS energy-dispersive X-ray spectroscopy (EDS) analysis was carried out to determine the quantitative composition of the samples. The SWIN Hall 8800 Hall Effect test device measured the carrier concentration and mobility. The electrical and optoelectronic measurements were performed using a Sourcemeter and a 365 nm UV light lamp.

## 3. Results and Discussions

### 3.1. Growth Per Cycle (GPC)

**Figure 1:**
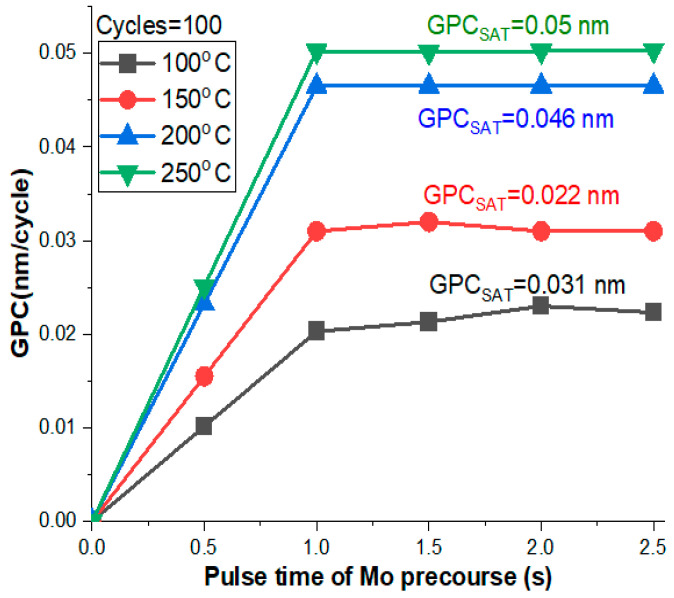
The GPC curves of the prepared samples at different deposition temperatures of 100, 150, 200, and 250 °C while keeping the number of cycles constant at 100.

The growth per cycle (GPC) curves of our deposited films via ALD typically exhibited a self-limiting growth behavior due to the saturation nature of the process as shown in Figure 1. The thickness of the deposited film grew linearly with the number of ALD cycles until a certain point where the growth rate slowed down and ultimately ceased. This was because the reactive sites on the substrate surface became fully covered with the deposited material and no further growth could occur [33]. Similar results of preparation of MoO_3_ using ALD were reported [32].

### 3.2. Surface Morphology of the Ultrathin Films

**Figure 2:**
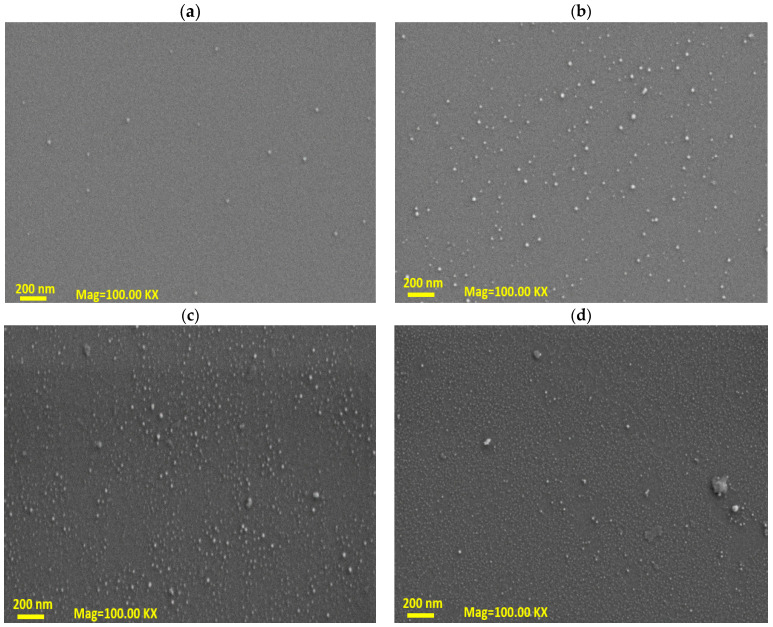
The FESEM of MoO_3_ samples at 100 KX magnification: (**a**) S1, (**b**) S2, (**c**) S3, and (**d**) S4. We chose a high magnification scale of 100 KX for better resolutions of the ultrathin films as reported in this study.

The film thickness was measured with a Filmetrics Analyzer. The measurements showed that the final S1, S2, S3, and S4 films had a thickness of 2.03, 3.1, 4.65, and 5.21 nm, respectively. One of the advantages of using the ALD system for preparing thin films is the forming of an ultrasmooth, flat, and homogenous film with a very small roughness value. To understand this behavior, we performed FESEM characterization to check the morphology of these samples. Figure 2 shows the FESEM morphology of the S1, S2, S3, and S4 samples at 100, 150, 200, and 250 °C, respectively. It seems that at a low deposition temperature of 100 °C, the films showed a highly flat surface with very small particles on the surface, similar to that reported before tungsten trioxide [34]. However, with increasing temperatures, small particles started to appear, representing a small agglomeration for the MoO_3_ sample. For sample (d), there were small contaminations that probably came from the chamber wall [15]. These findings made the produced thin films suitable for ultrathin, wafer-scale productions, and industrial applications [35]. Meanwhile, homogenous, smooth, and low roughness sources made the light scattering weaker and increased the light absorption as well [36]. Such advantages supported the ALD fabrication methods for the photodetector and electronic applications as reported here [37].

### 3.3. EDS Characterization

**Figure 3:**
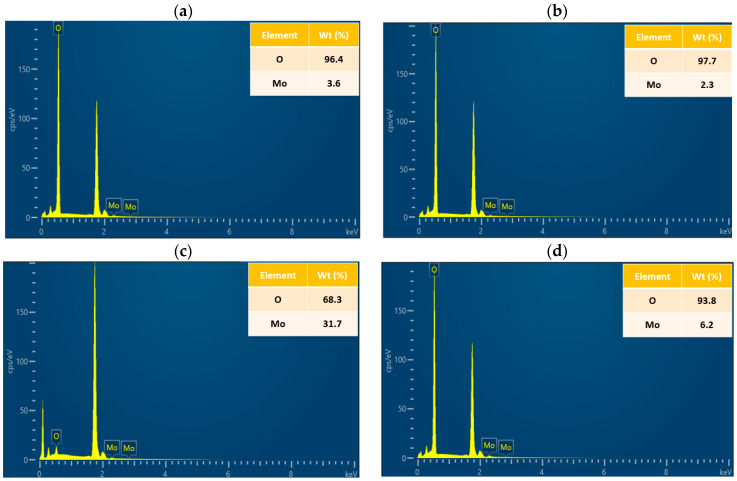
The EDS of MoO_3_/Si samples: (**a**) S1, (**b**) S2, (**c**) S3, and (**d**) S4. The Si peak was ignored in the EDS results because it had almost 99.1% which could disappear the Mo peaks. So, it was hard to detect Mo peaks in the presence of Si, due to the fact that the thickness of the Mo layer was a few nanometers.

EDS performed through an FESEM is a widely used technique in the field of materials science and engineering. It provides information about the elemental composition of a sample, including the concentration of each element present. EDS can provide information on both the surface and subsurface layers of the MoO_3_/Si thin film deposited by ALD, which is critical for understanding the film’s composition and structure. Many reported ALD-based studies used EDS for better characterizations such as WO_3_ [34], rhodium–iridium [38], TiO_2_ [39], and Al_2_O_3_ [40]. Figure 3 shows the collected EDS spectra from the samples after the post-annealing process for ensuring better crystallinity [12]. The concentrations of the O K and Mo L series elements were important for predicting the electrical and optoelectronic performances of the samples. The results here confirmed the presence of Mo and O elements with different concentrations based on the deposition temperatures. By looking at the O and Mo concentrations, we see that the sample at 200 °C showed higher concentrations of 31.7% Mo than the others, as shown in Figure 2c. This was followed by a weaker concentration of 6.2% Mo at 250 °C. The results confirmed the presence of Mo–O at higher concentrations at 200 °C which could confirm its higher and denser particle formations, and consequently higher electric and electronic properties as shown in the electronic results below. Moreover, the O was higher than the Mo content in all samples as expected for the MoO_3_ structure. In addition, some O from the Si substrate was attributed.

### 3.4. Surface Topography and Roughness

**Figure 4:**
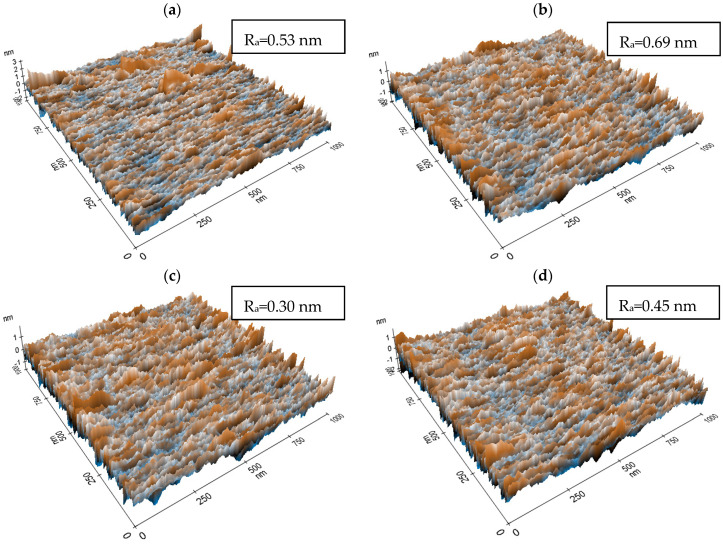
AFM topography and roughness of (**a**) S1, (**b**) S2, (**c**) S3, and (**d**) S4 where the average roughness is (R_a_).

AFM is a powerful technique that can be used to study the surface topography and roughness of MoO_3_ thin films. The technique works by using a cantilever with a sharp tip to scan the surface of the material and measure the deflection of the cantilever in response to the forces between the tip and the surface. AFM can provide high-resolution images of the surface topography of MoO_3_ thin films, including information on the shape and size of the surface features such as hills, valleys, and roughness. This information can be used to understand the morphological and textural characteristics of the material, which can be important for optimizing its use in various applications. The surface topography of samples S1, S2, S3, and S4 is reported in Figure 4. The figures show that all samples showed exceptional uniformity, thickness control, high surface quality, and low roughness as expected using ALD. This is because one of the key benefits of using ALD for producing MoO_3_ thin films is the capability of depositing a conformal coating with consistent thickness over complex surface geometries. This was achievable because of the self-limiting reaction mechanism utilized in ALD, which gives greater control over the deposition rate and uniformity of the material. The roughness was about 0.30 nm for the 200 °C as the lowest value and a higher value of 0.69 nm was attributed to the S2 sample. Similar results are reported for MoO_3_ by ALD [41]. Another study deposited MoO_3_ via ALD and reported a 0.68 nm roughness for 150 pulses [12].

## 4. Electrical Properties

### 4.1. Surface Resistance

**Figure 5:**
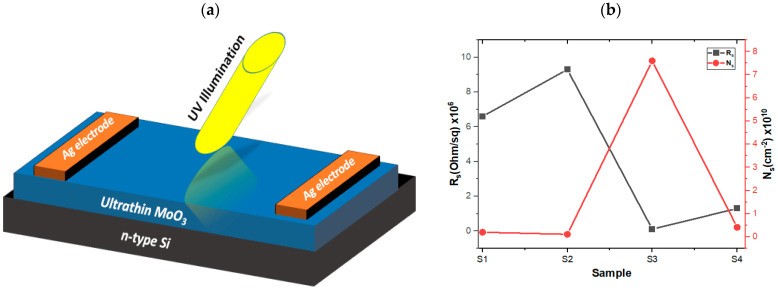
A shamanic diagram representing the fabricated device (**a**) and the relation between R_s_ and N_s_ of MoO_3_/Si of the S1, S2, S3, and S4 samples (**b**).

The four-probe measurement system was utilized to collect data at 300 k with an applied magnetic field of 7210 G for the fabricated device (Figure 5a). The results show that the samples showed *n*-type semiconducting behavior. This is may have come from the *n*-type nature of MoO_3_ and the presence of oxygen vacancies in suboxides [42]. The stoichiometric form of α-MoO_3_ is an insulator, but it is commonly found as an *n*-type semiconductor because of the presence of oxygen vacancies [43]. The manipulation of these vacancies in α-MoO_3_ has been previously utilized to adjust its chemical and electronic characteristics [27,43]. When annealed in air, there is a reduction in the electron concentration due to significant oxygen adsorption [44]. We measured the sheet resistance (R_s_), and sheet carrier concentration (N_s_) and reported them in Table 1. The behavior of the sheet resistance and sheet carrier concentrations of the S1, S2, S3, and S4 are reported in Figure 5b. Sample S3 showed the highest value of N_s_; in contrast, the lowest value was attributed to S2 as shown. This result shows that the S3 sample showed a higher conductivity and consequently a higher electric and optoelectronic response. This result supports the high electronic and photodetector properties of S3-200 °C under UV illuminations as reported below.

### 4.2. Current–Voltage Characteristics

**Figure 6:**
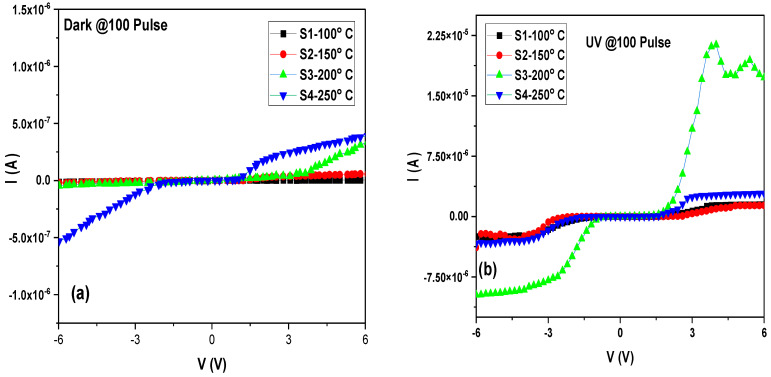
I–V characteristic of MoO_3_/Si heterostructure photodetector under dark (**a**) and UV irradiance (**b**) of the S1, S2, S3, and S4 samples.

The current–voltage (I–V) characteristics of the MoO_3_/*n*-Si heterostructure photodetector were explained according to the thermionic emission theory where [45]:

(1) $$I=I_{0}[exp\frac{q\left(V-IR_{s}\right)}{nkT}]$$

where *I*_0_ is the saturation current which is given by

(2) $$I_{0}=AA^{*}T^{2}[exp\frac{-q\varphi_{b}}{kT}]$$

and *n* is the ideality factor, q is the electron charge, V is the bias voltage, *R_s_* is the series resistance, k is the Boltzmann constant, T is the temperature in Kelvin, A is the active area, and A* is the Richardson constant (A* = 114 and 112 A.cm^−2^ K^−2^ for *p*- and *n*-type Si, respectively), Φ_b_ is the barrier height [46,47,48,49]. The I–V characteristics of the MoO_3_/*n*-Si heterostructure photodetector were measured at room temperature under the dark and UV light irradiance of 100 W/m^2^. Figure 6 shows the I–V plot for S1, S2, S3, and S4 samples in dark and UV illumination. Under UV illumination, the forward and reverse saturation currents were found to increase significantly, particularly for sample S3, which was prepared at a temperature of 200 °C. This could be attributed to the excellent carrier properties, very smooth surface, and high EDS content of the sample, which made it more resilient and stable under both the dark and UV conditions. In other words, the unique properties of sample S3 made it more responsive to UV light, resulting in higher levels of current flow in both the forward and reverse directions.

**Figure 7:**
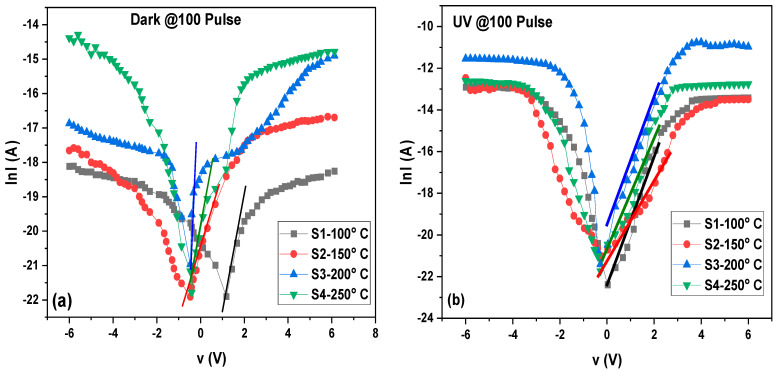
Semi-logarithmic curve of MoO_3_/Si heterostructure photodetector under dark (**a**) and UV irradiance (**b**) of the S1, S2, S3, and S4 samples.

Figure 7 shows the semi-logarithmic curve of the MoO_3_/Si heterostructure photodetector under dark and UV irradiance where the linear part followed ohms law. As the voltages increased, the current increased to a point where the relation collapsed. Both I_o_ and *n* were determined from the intercept and slope of the straight plot of the semi-logarithmic plot ln(*I*) vs. V of Figure 7. Additionally, the series resistance R_s_ represents the voltage drop across the photodetector and can be obtained throughout the relation [50]:

(3) $$\left(\frac{dV}{dlnI}\right)=\frac{nkT}{q}+IR_{s}$$

**Figure 8:**
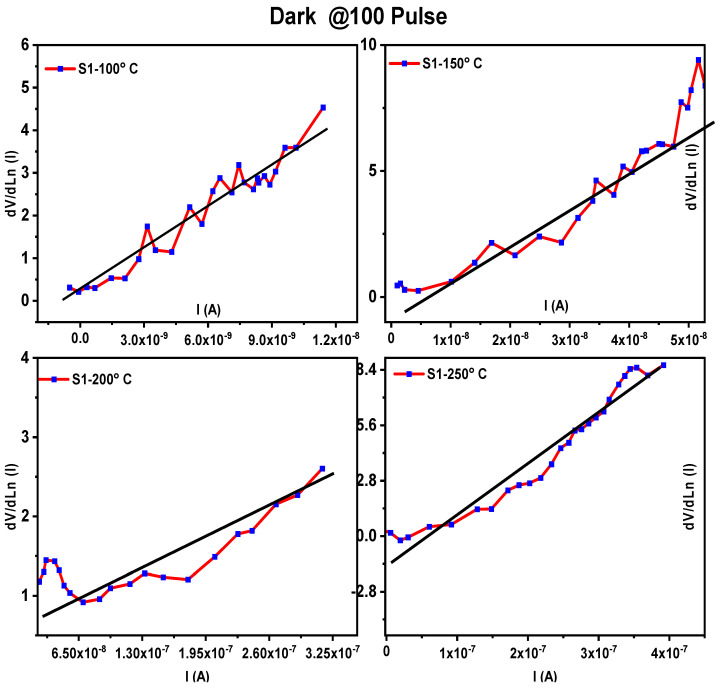
Plots of dV/dlnI vs. I of MoO_3_/Si heterostructure photodetector under dark illumination for different thicknesses.

**Figure 9:**
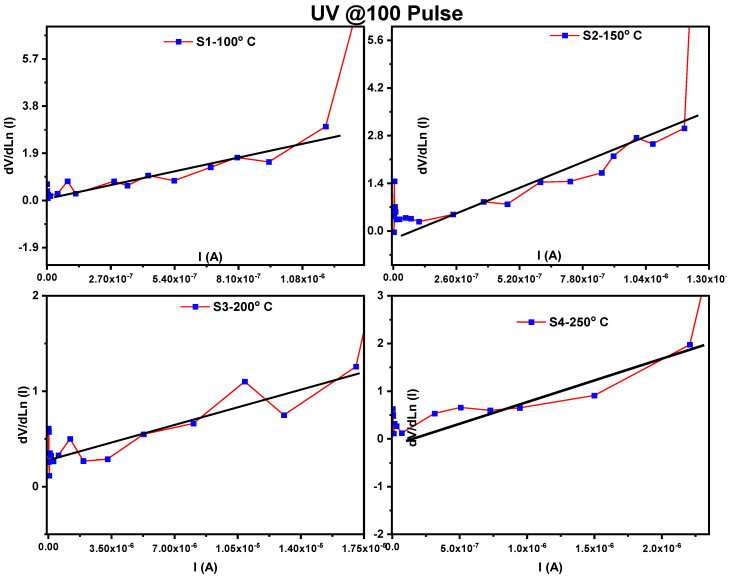
Plots of dV/dlnI vs. I of MoO_3_/Si heterostructure photodetector under UV illumination for different thicknesses.

Figure 8 and Figure 9 display dV/dln (I) versus I for the MoO_3_/Si heterostructure photodetector under dark and UV illumination, respectively. By plotting dV/dln(I) against the current (I), the slope of the resulting curve represented the R_s_ value while the intercept on the y-axis corresponded to nkT/q, as per Equation (3). The values of R_s_ and the ideality factor (*n*) are listed in Table 2 and Table 3 for the dark and UV illumination, respectively.

An alternative method to extract the ideality factor (*n*), series resistance (R_s_), and barrier height (Φ_b_) has been proposed by Cheung [51]. According to this method, H(I) is defined as the Cheung function and is given by,

(4) $$H\left(I\right)=V-n(\frac{KT}{q})Ln(\frac{I}{AA^{*}T^{2}})$$

where H(I) is given as,

(5) $$H\left(I\right)=n\varphi_{b}+IR_{s}$$

**Figure 10:**
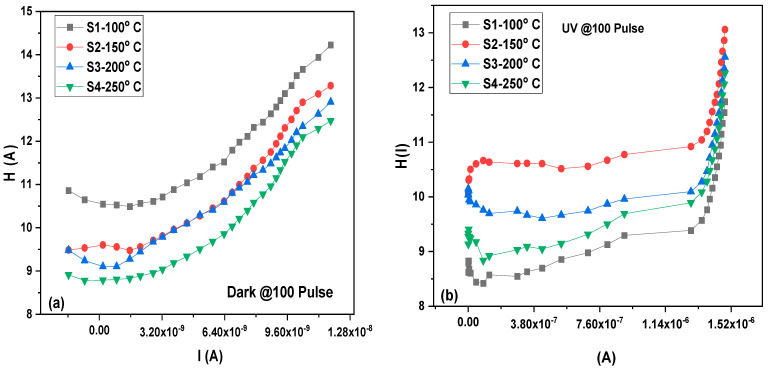
H(I) vs. I of MoO_3_/Si heterostructure photodetector at different irradiance levels for dark (**a**) and UV irradiance (**b**).

Figure 10 shows the plot of H(I) verse I, providing a straight line with an intercept equal to nΦ_b_, while the slope gives the series resistance (R_s_). Table 2 and Table 3 list and tabulate the output parameters of the barrier height (Φ_0_) and series resistance (R_s_) for the dark and UV illumination conditions, respectively.

**Figure 11:**
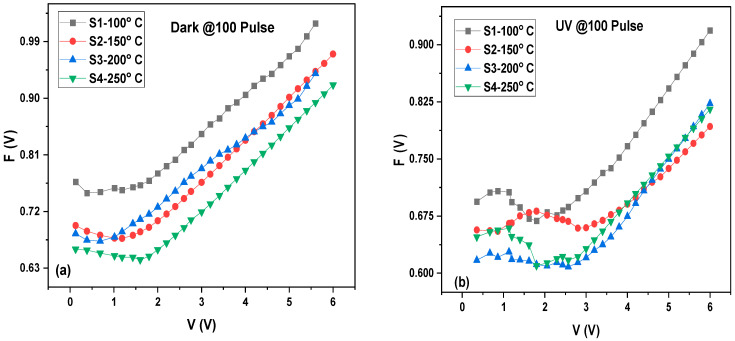
F (V) vs. Voltage of MoO_3_/Si heterostructure photodetector at different depositing temperatures for dark (**a**) and UV irradiance (**b**).

An alternative method proposed by Nord was used to compare the barrier height and series resistance (R_s_) of the MoO_3_/Si heterostructure [52]. The Norde function F(V) against V is a tool used to study the behavior of a photodetector at different deposition temperatures. According to this method, the Nord function *F*(*V*) is defined as:

(6) $$F\left(V\right)=\frac{V}{ϒ}-\frac{1}{\beta }Ln(\frac{I}{AA^{*}T^{2}})$$

where β=q/KT and ϒ is ≥ the ideality factor (*n*). The active barrier height is given by,

(7) $$\varphi_{b}=F\left(V_{min}\right)+\frac{V_{min}}{ϒ}-\frac{1}{\beta }$$

where *F*(*V_min_*) is the minimum value of *F*(*V*) corresponding to *V_min_*.

(8) $$R_{s}=\frac{ϒ-n}{\beta I_{min}}$$

where (I_min_) is the current value corresponding to the minimum voltage value (V_min_). Figure 11 shows the Norde function F(V) against V for the MoO_3_/Si heterostructure photodetector at a different level of illumination.

**Figure 12:**
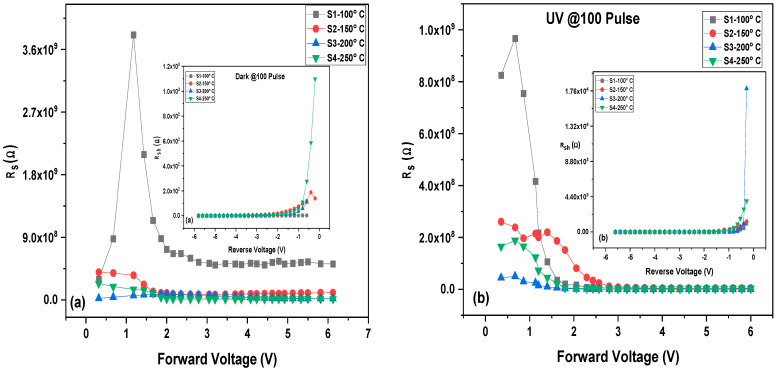
Series resistance and inset shunt resistance of MoO_3_/Si at dark (**a**) and UV illumination (**b**).

The barrier height, series resistance, and ideality factor (*n*) for the MoO_3_/Si heterostructure photodetector were calculated and are presented in Table 1 and Table 2. The observed variation in the ideality factor (*n*) can be attributed to the effects of the series resistance, which causes a voltage drop across the interfacial thin film and the charge of the interface states with the bias in the low voltage region of the I–V diagram. Both the R_s_ and R_sh_ values have considerable influence on many electronic devices. The experimental R_s_ and R_sh_ values are determined by plotting the structure resistance (R_i_) vs. applied bias voltage (V) as shown in Figure 12. It has been observed that at a high forward bias voltage, the R_s_ designates a nearly constant value for positive voltages greater than 0.5 V for all thicknesses. Subsequently, at a negative bias voltage, the value of R_i_ becomes constant for all thicknesses that correspond to R_sh_. The transportation of charge carriers through the diffusion layer or the enhanced tunneling current through the barrier high using heavy-doped *n*-type Si can cause a lower value for the series resistance R_s_ [53].

## 5. Optoelectronic Properties of MoO_3_/Si under UV

**Figure 13:**
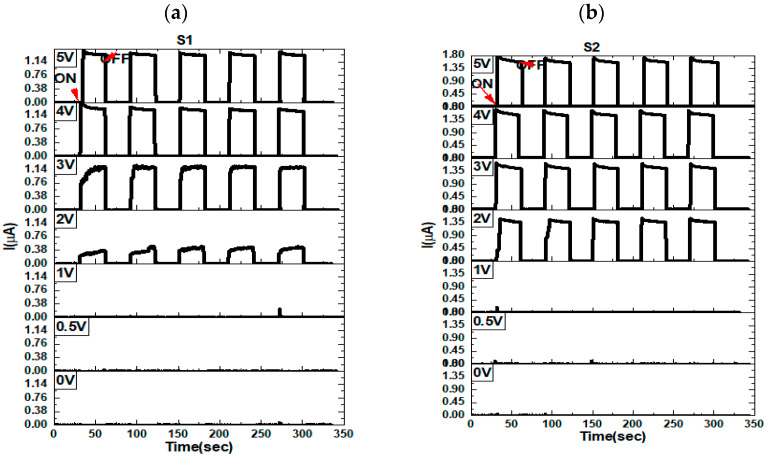

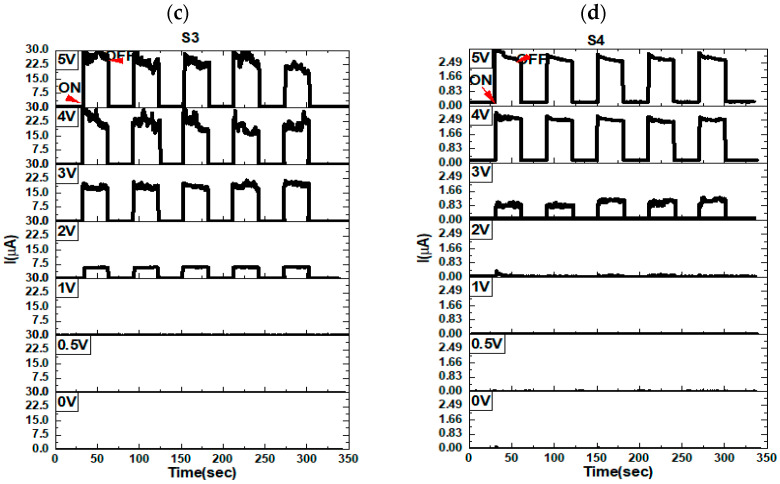
The photodetector ON–OFF performances with time at different applied voltage biases of 0, 0.5, 1, 2, 3, 4, and 5 V for S1 (**a**), S2 (**b**), S3 (**c**), and S4 (**d**) samples. Where (**a**–**d**) are deposited at 100, 150, 200, and 250 °C, respectively. All these samples were deposited in 100 pulse conditions.

The optoelectronic properties of a material refer to the combination of its electrical and optical characteristics that are important in the operation of optoelectronic devices. In the case of a MoO_3_/Si heterostructure under UV illumination, these properties can be evaluated through parameters such as the photocurrent, ON–OFF response, and photocurrent–time behaviors. These parameters determine the ability of the heterostructure to convert light into electrical signals, and thus play a critical role in its use as a photodetector. By examining the optoelectronic behavior of MoO_3_/Si under UV light, researchers aim to gain a better understanding of the mechanisms behind the light-to-electricity conversion, which can be useful in developing more effective and efficient photodetector devices as addressed here. By considering the electronic properties and the effect of UV illuminating on the MoO_3_/Si structure, a further investigation here is covered for the photo response of it with time. We deeply illustrated the effect of the applied voltages of 0, 0.5, 1, 2, 3, 4, and 5 V on the samples S1, S2, S3, S4, and S5. The ON–OFF dynamic of the samples under UV illuminations and dark conditions for 0, 0.5, 1, 2, 3, 4, and 5 V biases are demonstrated in Figure 13. Generally, the photocurrent was increasing with the bias voltage for different maximum currents as shown. We kept the upper limit of each curve constant based on the highest generated photocurrent of 5 V for better comparison. All the samples had good stability with time and with high photocurrent upon illuminations and low current under dark conditions. We saw that S3 had a better photocurrent than the other samples up to 30 µA at 5 V followed by the S4 sample with a large gap. This enhancement in the photocurrent was attributed to the high I–V curve and high electron density as recommended by the Hall effect measurements and EDS results. However, S1 and S2 had almost similar behaviors with a small enhancement in the case of S2 due to the low concentration of the Mo, as 100 and 150 °C were not optimum for the MoO_3_ deposition by ALD. For the case of S3, it started to observe a photocurrent of around 7 µA at 2 V while the samples did not excess 2 µA, indicating the good performance of the S3 sample. The *n*-type behavior was also attributed to these results.

**Figure 14:**
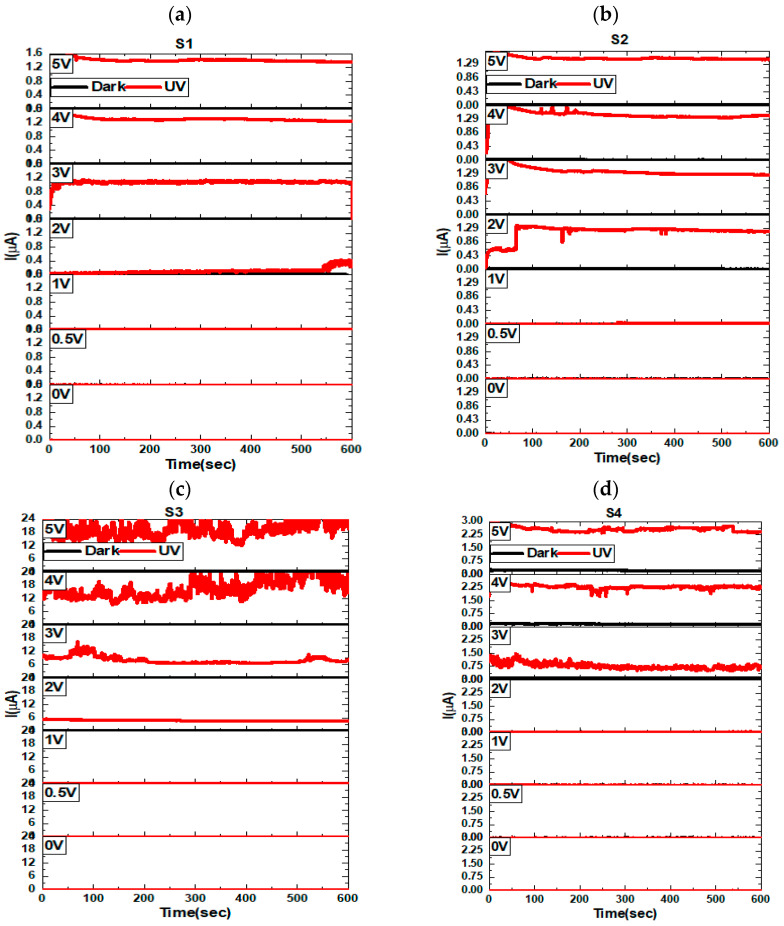
The photodetector performance with time at different applied voltage biases of 0, 0.5, 1, 2, 3, 4, and 5 V for S1 (**a**), S2 (**b**), S3 (**c**), and S4 (**d**) samples. Where (**a**–**d**) are deposited at 100, 150, 200, and 250 °C, respectively. All these samples were deposited via 100 pulse conditions.

**Figure 15:**
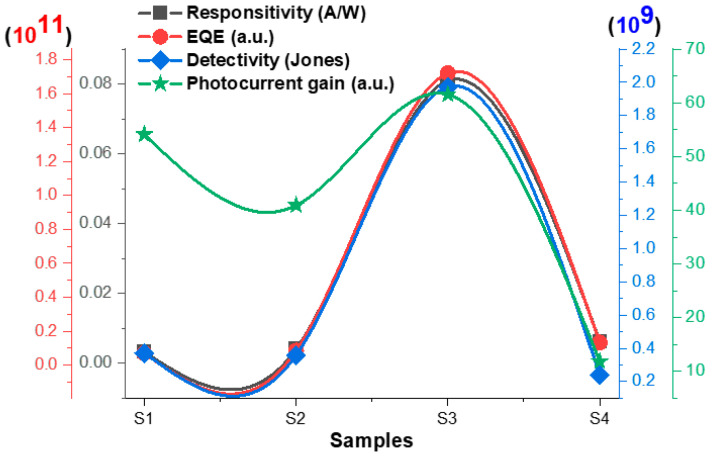
A relation between the responsitivity, EQE, detectivity, and photocurrent gain of each sample: S1, S2, S3, and S4.

In addition, we checked the stability of these samples under dark and UV light with time in static mode as shown in Figure 14. As was also expected here, the photocurrent was high in the S3 case. We observed that S3 became unstable at high applied bias voltages, so it was recommended for the ultrathin MoO_3_/Si photodetector here to work at low voltages and high voltages as well. Because a 5 V bias gave the sample more activations for better photoconductivity and a better photodetector response, we calculated the photocurrent gain, photoresponsivity ($R_{\lambda }$), external quantum efficiency (EQE), detectivity ($D^{*}$), and finally the response/recovery time at 5 V biase. The calculations were collected based on the previous reports [54,55,56]. For a UV photodetector, the responsivity is important as it is a measure of the sensitivity of a photodetector to light. The photocurrent gain is a measure of the ability of a photodetector to amplify the small electrical signal generated by the incident light to produce a larger output signal. While the EQE provides us with information about the efficiency of a photodetector at converting photons into electrons, the detectivity is also important for measuring the ability of a photodetector to detect very weak signals in the presence of noise. The results of the responsitivity, EQE, detectivily, and photocurrent gain of each sample of S1, S2, S3, and S4 are represented in Figure 15. The general behavior of these parameters confirming the same results of the electronic and optoelectronic performance of S3 was the best compared to the other samples, with an EQE of 1.7 × 10^11^, and responsitivity of 81 mA/W. These results show that the effect of a few-nanometer-thick MoO_3_ deposited by ALD on a Si substrate showed an interesting UV optoelectronic performance.

**Figure 16:**
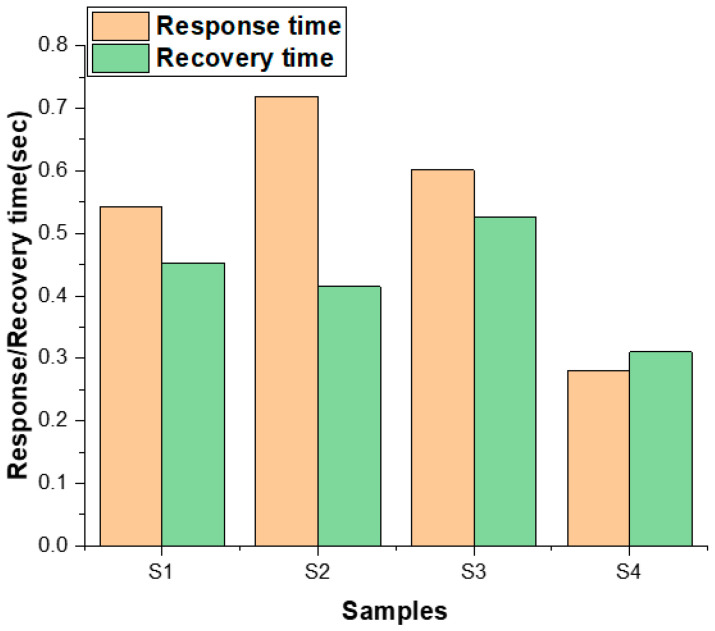
The response and recovery time of each sample: S1, S2, S3, and S4.

Interestingly, the samples show a very fast response and recovery time at 5 V as shown in Figure 16. The response and recovery time of a photodetector are important factors to consider when selecting a detector for a particular application, as they can impact the accuracy and speed of the measurements. The response time of a photodetector refers to how quickly it can respond to changes in the incident light, while the recovery time refers to how quickly it can return to its baseline state after the light is removed. A fast response time is important in applications where rapid changes in light intensity need to be detected, such as in optical communication systems or high-speed imaging. A fast recovery time is important in applications where the photodetector needs to be able to detect rapidly changing light signals with minimal lag or distortion. The results show that the response time and recovery time are comparable and near each other. These values are in the range of 0.5 s.

We compared our results with the current studies involving MoO_3_. Since MoO_3_ has several desirable properties, including a wide bandgap, high carrier mobility, stability, and in-plane anisotropy, these make it a promising material for the production of advanced UV photodetectors [21]. Mendoza et al., were the first to create a large-scale MoO_3_-based photodetector using a 1.4–6 nm thick MoO_3_ layer produced through van der Waals epitaxy on muscovite mica, which was then transferred to a SiO_2_ substrate. The resulting field effect device had a responsivity of 30 mA W^−1^ and a slow response time of approximately 20 s at a 405 nm wavelength [22]. However, for our device which was fabricated using ALD, we had a better responsivity of 80 mA W^-^ and a fast response time of 0.6 s for the S3 sample, which was deposited at 200 °C. In a study conducted by Li et al., a UV photodetector was created using centimeter-scale single crystals of MoO_3_ that were grown using PVD. The photodetector displayed a responsivity of 65.6 AW^−1^ when subjected to 405 nm light after undergoing vacuum annealing for 2 h. The improved mobility of the photogenerated carriers that occurred post-annealing resulted in a faster response time of just 2 s [23]. However, for our case, our photodetector was still more promising besides the annealing process in air, and for a fast period of 15 min. In a separate study by Arash et al., highly efficient UV photodetectors were fabricated on non-stoichiometric MoO_3−X_ layers. The introduction of oxygen vacancies resulted in Mo^5+^ states that were partially reduced and acted as traps for photogenerated electrons, facilitating a charge carrier separation. This resulted in a high responsivity of 54.4 AW^−1^ being observed at 365 nm. It is worth noting that trap states were still present despite these findings [24]. In addition, this result is still beyond our simple MoO_3_/Si photodetectors via the ALD deposition system.

In summary, this study aimed to investigate the electronic properties and the effect of UV illumination on the MoO_3_/Si heterostructure. Through the analysis of the photocurrent and thermionic emission response, we were able to demonstrate the ON–OFF dynamic behavior of the samples under different bias voltages and conditions. The results showed that sample S3 had the highest photocurrent, followed by sample S4, while samples S1 and S2 had similar behaviors. Additionally, the stability of the samples under dark and UV light conditions was also analyzed, and it was found that sample S3 had the highest photocurrent but became less stable at higher applied bias voltages. Based on these findings, it is recommended that an ultrathin MoO_3_/Si photodetector operate at low voltages for better efficiency.

## 6. Conclusions

In conclusion, ultrathin films of MoO_3_ have emerged as a promising material in the field of electrical and optoelectronic applications, owing to their remarkable properties, such as a high surface area, electrical conductivity, and optical transparency. This study demonstrated that films of MoO_3_/Si, prepared by atomic layer deposition with thicknesses of a few nanometers, exhibit exceptional reactivity, making them an ideal candidate for compact electronic devices and highly reactive surfaces. Furthermore, the study revealed that ultrasmooth films of less than 1 nm roughness were effective in achieving a high-performance electric and photodetector performance at low applied bias voltages. The deposition temperature also played a significant role in determining the surface electronic carriers, with the results indicating that 200 °C yielded the highest electronic carrier density with an EQE of 1.7 × 10^11^ and responsitivity of 81 mA/W. The I–V characteristics were analyzed using thermionic emission theory, while Cheung and Nord’s methods were utilized to determine the electronic parameters of the photodetector. The consistency of the results obtained from these methods confirms the accuracy of the findings. However, the higher ideality factor values suggest the presence of series resistance, which caused a voltage drop across the interfacial thin film and charge accumulation at the interface states between the MoO_3_ and Si surface in the low voltage region of the I–V diagram. Overall, these results indicate that ultrathin films of MoO_3_ hold great promise for developing advanced electronic and optoelectronic devices, and further research is warranted to explore their full potential.

## Figures and Tables

**Table 1:** Shows the surface sheet resistance (R_s_) and sheet carrier concentration (N_s_).

Samples	R_s_ (Ohm/sq)	Type	N_s_ (cm^−2^)
S1	6.6 × 10^6^	N	2.0 × 10^9^
S2	9.3 × 10^6^	N	1.1 × 10^9^
S3	1.1 × 10^5^	N	7.6 × 10^10^
S4	1.3 × 10^6^	N	4.1 × 10^9^

**Table 2:** The ideality factor, barrier height, and series resistance of MoO_3_/Si in the dark were determined through various methods at different preparation temperatures.

Under Dark											
Samples	(TE)			Cheung (H)			dV/dlnI		Nord (F)		
	*n*	$\Phi_{b}eV$	$R_{s}\Omega$	*n*	$\Phi_{b}eV$	$R_{s}\Omega$	*n*	$R_{s}\Omega$	*n*	$\Phi_{b}eV$	$R_{s}\Omega$
**S1-100 °C**	13.5	0.67	2.98 × 10^8^	13.6	0.73	3.30 × 10^8^	16.4	4.45 × 10^8^	13.5	0.66	1.26 × 10^7^
**S2-150 °C**	13.7	0.68	7.46 × 10^7^	13.1	0.64	7.70 × 10^7^	11.9	4.17 × 10^7^	13.7	0.67	7.84 × 10^6^
**S1-200 °C**	13.3	0.58	1.81 × 10^7^	15.3	0.71	1.30 × 10^7^	14.2	1.89 × 10^7^	13.6	0.60	3.5 × 10^5^
**S1-250 °C**	13.6	0.66	1.28 × 10^7^	13.4	0.63	9.15 × 10^6^	13.4	2.00 × 10^7^	13.7	0.65	4.67 × 10^6^

**Table 3:** The calculated ideality factor, barrier height, and series resistance of MoO_3_/Si under UV illumination at different preparation temperatures were obtained from the various methods.

Under UV Illumination											
Samples	(TE)			Cheung (H)			dV/dlnI		Nord (F)		
	*n*	$\Phi_{b}eV$	$R_{s}\Omega$	*n*	$\Phi_{b}eV$	$R_{s}\Omega$	*n*	$R_{s}\Omega$	*n*	$\Phi_{b}eV$	$R_{s}\Omega$
**S1-100 °C**	11.9	0.71	2.8 × 10^6^	12.1	0.70	1.47 × 10^6^	12.6	1.38 × 10^6^	11.7	0.67	6.75 × 10^7^
**S2-150 °C**	16.8	0.60	3.51 × 10^6^	14.8	0.70	1.34 × 10^6^	14.8	1.1 × 10^6^	14.8	0.64	1.1 × 10^6^
**S1-200 °C**	12.8	0.58	1.81 × 10^5^	12.8	0.63	1.85 × 10^5^	15.8	6.0 × 10^5^	15.9	0.62	6.0 × 10^5^
**S1-250 °C**	15.7	0.68	1.23 × 10^6^	13.6	0.65	6.5 × 10^5^	13.6	13.6 × 10^5^	13.4	0.64	3.6 × 10^5^

## Data Availability

The data presented in this study are available on request from the corresponding authors.
